# A new method to identifying optimal adjustment strategy when the car cockpit is uncomfortable: optimal state distance method

**DOI:** 10.7717/peerj-cs.1324

**Published:** 2023-04-06

**Authors:** Fei Chen, Hongbo Shi, Jianjun Yang, Yu Lai, Jiahao Han, Yimeng Chen

**Affiliations:** 1 School of Automobile and Transportation, Xihua University, Chengdu, Sichuan Province, China; 2 Vehicle Measurement, Control and Safety Key Laboratory of Sichuan Province, Xihua University, Chengdu, China; 3 Provincial Engineering Research Center for New Energy Vehicle Intelligent Control and Simulation Test Technology of Sichuan, Xihua University, Chengdu, China; 4 School of Mechanical Engineering, Xihua University, Chengdu, Sichuan Province, China

**Keywords:** Identify, Comprehensive evaluation, Comfort adjustment, The optimal strategy, Optimal state distance

## Abstract

With the rapid development of the automobile industry, the comfort of the cockpit has become the standard for judging the quality of the car. People have also put forward higher requirements for cockpit comfort. In the process of driving, the cockpit environment will constantly change, and the comfort will also change. When the comprehensive comfort level of the cockpit decreases and the occupants feel uncomfortable, the cockpit comfort should be adjusted. In this article, a cockpit comfort evaluation model is established to realize the evaluation of cockpit comfort. In addition, we elaborate the theory of optimal state distance, where the numerical magnitude of the optimal state distance is used to reflect the extent to which an indicator deviates from its optimal state. Also, a cockpit optimal adjustment strategy identification model is established based on the theory, which can obtain the optimal adjustment strategy in a certain cockpit operating environment, facilitate the timely adjustment of the corresponding actuator, and realize the dynamic monitoring and adjustment of cockpit comfort. This project provides a reference direction for cockpit comfort adjustment, which is of great significance for future research and development of automotive cockpit comfort.

## Introduction

More and more Chinese are using passenger cars to commute to work. During short commutes, it is critical to quickly provide acceptable cockpit comfort so that the driver is more focused and alert (Norin & Wyon, 1992). In the future, people will spend more time and energy in the cockpit of the car, doing more than driving, such as learning, entertainment, *etc.* (Sun, Cao & Tang, 2021). The car cockpit has gradually become an intelligent interactive environment, developing towards a third space (Yang et al., 2022a; Yang et al., 2022b). However, inadequate cockpit comfort can lead to human dissatisfaction and have negative effects on productivity and performance (Budaiwi, 2007). It is clear that cockpit comfort is gradually becoming a criterion for evaluating the quality of cars. The study of cockpit comfort evaluation is also a very meaningful and valuable thing.

Moreover, it is worth noting that comfort studies are a popular interdisciplinary discipline. In regard to comfort studies, there are aircraft cockpits and high-speed rail cockpits (Cui et al., 2017; Liping et al., 2018; Liu, Yu & Chu, 2020; Chen et al., 2020; Wang et al., 2021), and indoor living environments (Oseland, 1995; Andargie, Touchie & O’Brien, 2019). Comfort evaluation is of interest in many areas. Therefore, the research on cockpit comfort in this article has great significance and value.

In the “Materials and Methods” section, the experiments and the model developed in this article are introduced. By conducting the cockpit comfort evaluation experiments, a cockpit comfort evaluation model is established, which can realize the comfort evaluation of the cockpit. By conducting cockpit comfort adjustment experiments, a new optimal state distance (OSD) theory proposed in this article is applied to establish a cockpit comfort optimal adjustment strategy identification model, which can identify the optimal adjustment strategy when the cockpit is uncomfortable and control the actuator for adjustment. In the “Results and Discussion” section, the model developed in this article is analyzed and discussed. The optimal adjustment strategy model based on the OSD theory proposed in this article is compared with several classical machine learning methods to verify that the model developed in this article has good accuracy and is of great value for engineering applications. In the “Conclusion” section of this article, the whole work is summarized. In the “Limitations and Future Work” section, we describe the limitations of the model and the future directions of the work.

## Literary Review

In recent years, much research has been conducted on the comfort of automobiles (Gkartzonikas & Gkritza, 2019). There are many factors that affect cockpit comfort, such as acoustic environment, optical environment, thermal environment, seat comfort, vehicle vibration, *etc*. (Demić, Lukić & Milić, 2002; Nor et al., 2008; Siefert, Pankoke & Wölfel, 2008; Nahvi, Fouladi & Nor, 2009; Szczurek & Maciejewska, 2016; Xu et al., 2022). Shek & Chan (2008) collect actual interior thermal and air parameter data, as well as subjective passenger satisfaction and perception votes, by taking cockpit physical parameter measurements and subjective questionnaires. By analyzing the correlation between subjective and objective data, a combined comfort model is established, which helps to assess passengers’ dissatisfaction with various sensory voting combinations of thermal comfort and air quality. Ali Böke et al. (2022) proposed a method for subjective and objective evaluation of vehicle ride comfort through road tests. Previati, Gobbi & Mastinu (2016) introduced the subjective and objective ride comfort evaluation of agricultural tractors and establish a comfort evaluation model that can quickly simulate the movement of the cab and evaluate its comfort performance. It is understood from previous literature that the current evaluation of comfort usually uses a combination of subjective and objective evaluation, (Da Silva, 2002; Naddeo, Cappetti & D’Oria, 2015) using data measurement and passenger scoring to obtain an evaluation model of cockpit comfort. In addition, previous studies of comfort have often been studies of single factors. Della & Romitelli (1993) evaluated the thermal comfort of the passenger compartment of the car. Zhou, Lai & Chen (2019) studied the thermal comfort of passenger cars under actual outdoor driving conditions. Da Silveira Brizon & Medeiros (2012) combined subjective and objective evaluation to study the evaluation of the acoustic comfort of motor vehicles. Wu, Liu & Pan (2008) showed a new car-following model focusing on passenger comfort, and established a braking comfort model for car-following according to the relationship between vehicle deceleration and occupant comfort. Starting from the acoustic environment, optical environment, and thermal environment that affect the comfort of the cockpit, this article carries out the cockpit comfort evaluation experiment, establishes the cockpit comfort evaluation model, and realizes the comprehensive comfort evaluation of a certain cockpit working environment.

While the car is moving, the cockpit environment is constantly changing, and so does the comfort. When the overall comfort of the cockpit is reduced and the occupants feel uncomfortable, the cockpit comfort needs to be adjusted. However, the main causes of cockpit micro-discomfort may be different, and how identifying the main causes of cockpit discomfort and adjusting them is the key to solving the problem. Support vector machines, XGBoost, and other methods in machine learning are widely used in identification problems. Sugumaran & Ramachandran (2007) used decision trees for roller-bearing troubleshooting. Liu et al. (2013) constructed a multi-fault classification model based on a support vector machine and is successfully applied to the bearing fault diagnosis of electric locomotives. Lin, Wei & Junjie (2019) proposed a method that combines deep convolutional neural networks (DCNN) with support vector machines (SVM) for the automatic identification of microseismic waveforms. Yoo et al. (2020) used decision trees to perform chest X-rays to diagnose COVID-19. Biddle & Fallah (2021) used SVM to detect and identify faults in the sensors of autonomous vehicle control systems. Ye et al. (2022) established a diagnostic model of OSA in children based on the XGBoost algorithm. Using heart rate and blood oxygen data as the main features, a machine learning diagnostic model based on the XGBoost algorithm can accurately identify children with OSA at different severities. Wang et al. (2023) applied the XGBoost algorithm to study the classification of seismic events occurring at local and regional distances and compared the performance of the SVM algorithm. However, machine learning methods require a lot of high-quality training data to ensure that the obtained model has a good recognition effect, and the acquisition of training data is often difficult.

In addition to machine learning methods, distance classifier methods are often used in such problems. Commonly used distances are Euclidean distance, Hamming distance, minimum distance, and Manhattan distance, *etc*., which are also widely used in classification, identification, and multi-attribute decision problems. S. Senda, Minoh & Katsuo (1995) Chinese character recognition with minimum distance. Pourhossein, Gharehpetian & Rahimpour (2011) use a Euclidean distance classifier to diagnose the buckling severity of transformer windings. Rai & Yadav (2014) propose a new method that combines a support vector machine and Hamming distance for identifying iris patterns. Joseph, George & Gaikwad (2020) use Euclidean distance and Manhattan distance to classify handwritten MODI scripts, respectively. Distance recognition has the characteristics of simple and fast operation and strong generalization ability, which has great value in engineering applications.

This article defines a new kind of distance: the optimal state distance (OS distance), which represents the distance at which an indicator deviates from the optimal state, and the larger the distance, the greater the parameter value of the indicator deviates from its optimal state. In the cockpit comfort adjustment, the larger the OS distance is the main indicator that affects the comfort of a certain cockpit environment. At the same time, this article defines the correction coefficient in the process of OS distance calculation, which can be weighted according to the needs of practical engineering applications to obtain the most realistic mathematical model. OS distance also has the characteristics of simple and fast operation, which is conducive to dynamic monitoring and adjustment of cockpit comfort and ensuring the occupants’ riding experience.

## Materials & Methods

### Establishment of cockpit comfort evaluation model

#### Car cockpit comfort evaluation system

**Figure 1:**
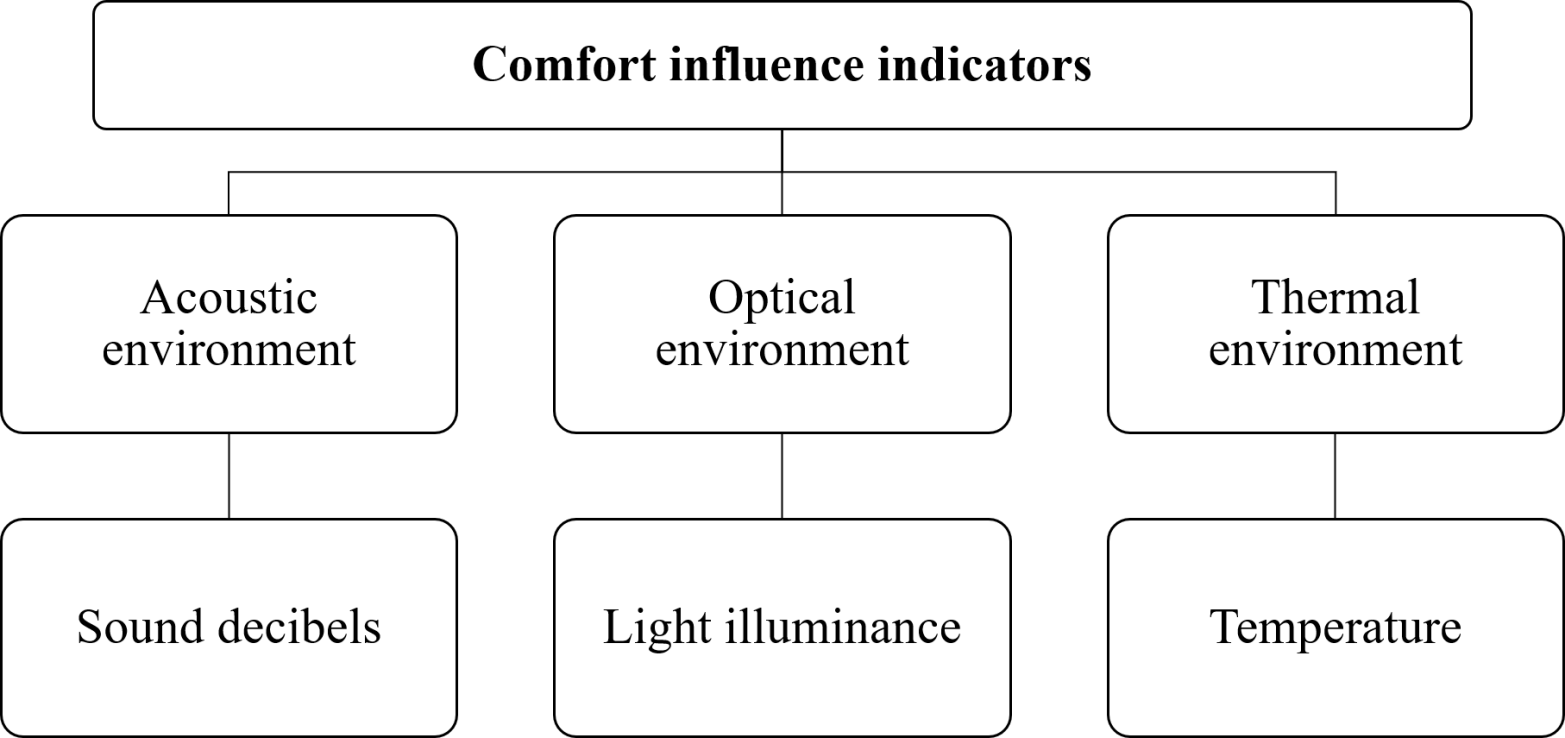
Comfort evaluation system for automobile cockpit.

After literature investigation and combined with expert guidance, the cockpit comfort is evaluated from three indicators of the acoustic environment, optical environment, and thermal environment that affect cockpit comfort, to establish the evaluation system of car cockpit comfort as shown in Fig. 1.

#### Cockpit comfort evaluation experiment

In this article, we conducted a cockpit comfort evaluation experiment and a cockpit comfort adjustment experiment. All experiments involved in this article have obtained the ethical certification of the Ethics Committee of the School of Automotive and Transportation of Xihua University (2021LL(01)).

**Figure 2:**
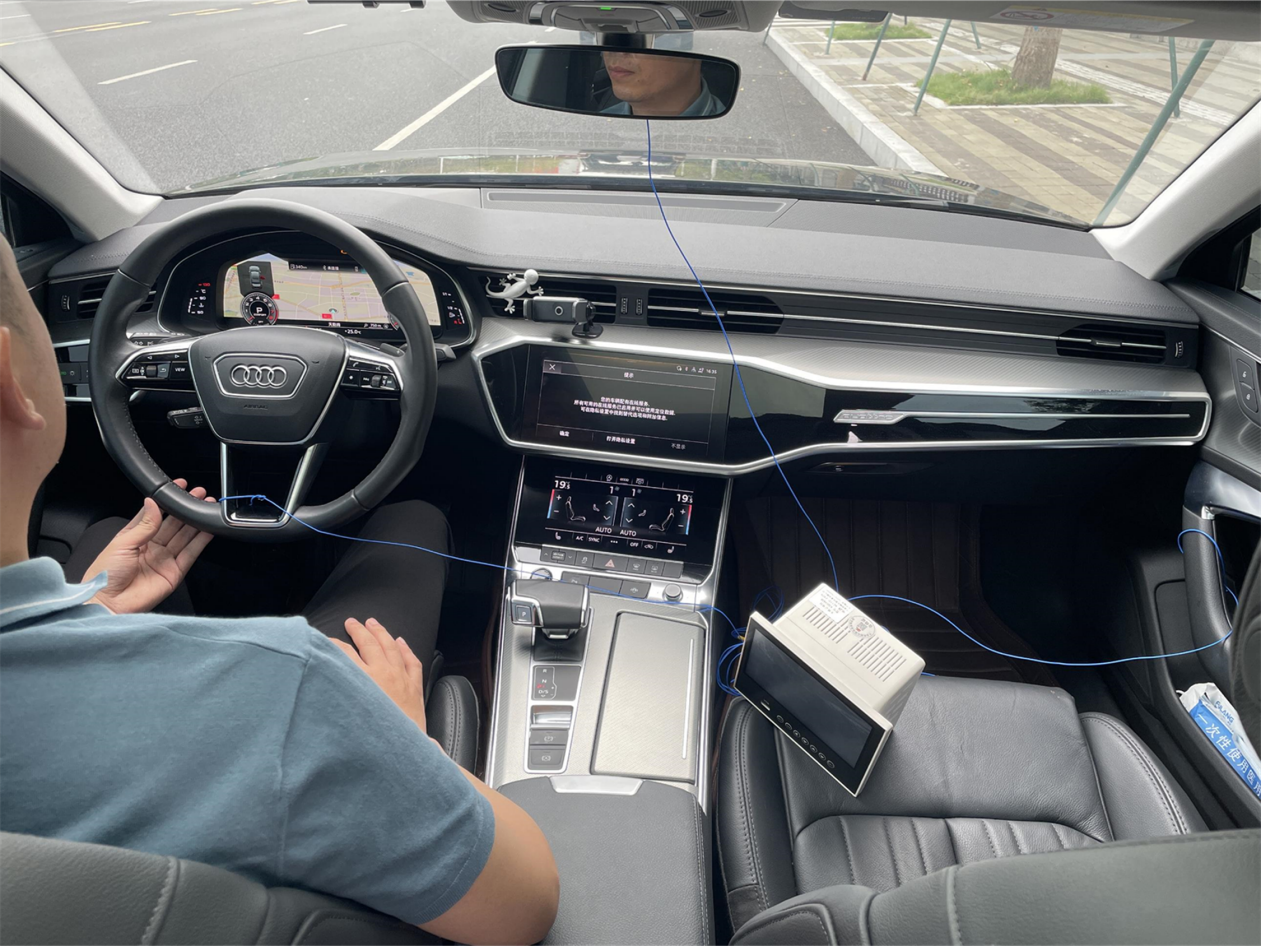
Cockpit environment test.

**Figure 3:**
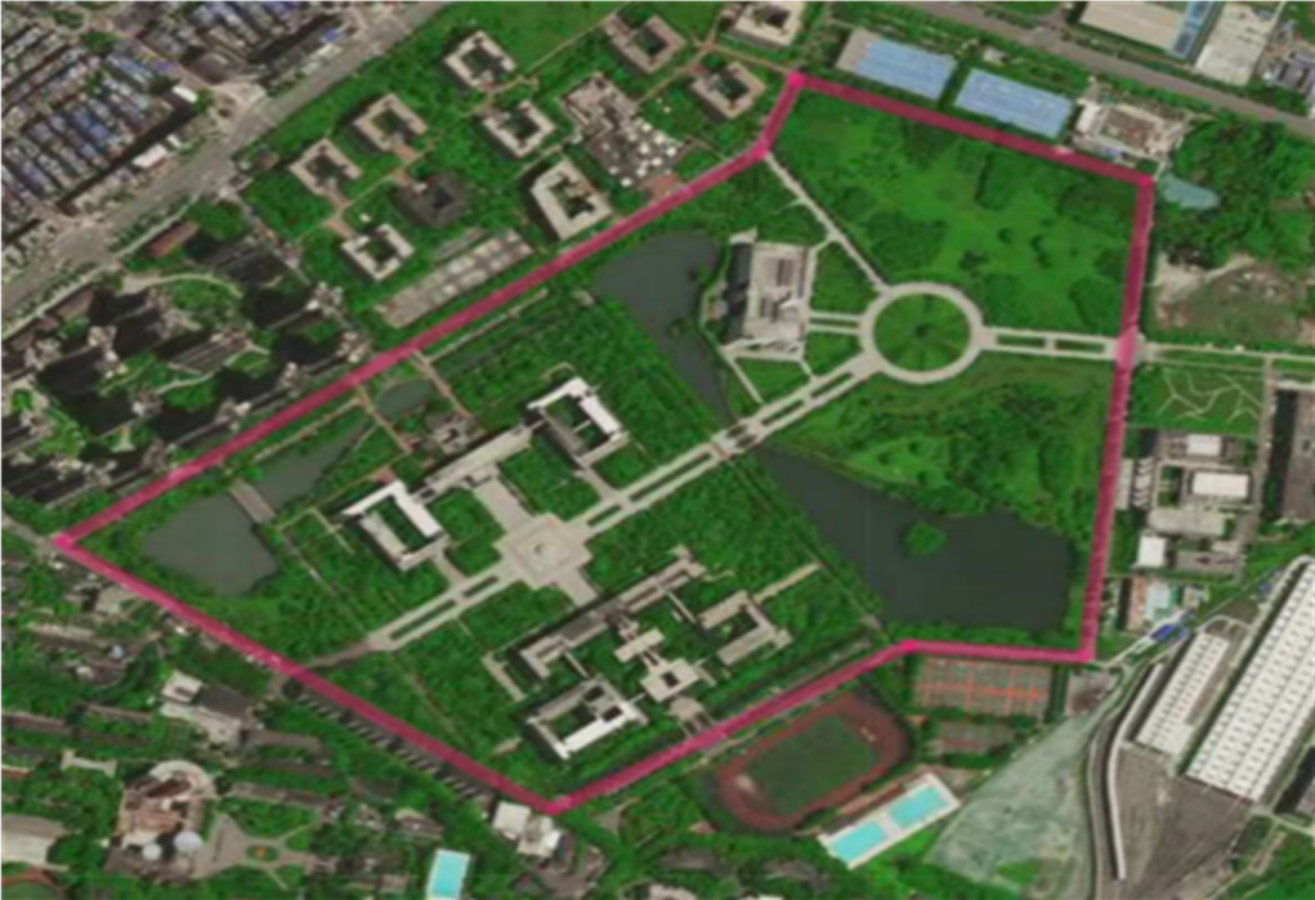
Specific route test.

In the cockpit comfort evaluation experiment, the Audi A6L (2021) was selected as the cockpit environment for experimental evaluation, as shown in Fig. 2. The experiment was carried out on the campus of Xihua University, and a road section was simulated as a real traffic line. The roadmap is shown in the red line in Fig. 3.

This experiment was conducted in sunny and windless weather. The outdoor temperature was approximately 33 degrees Celsius. Since constant speed is the most common driving condition in real life, the experimental conditions for this experiment were all set to constant speed, with the speed controlled at about 40 km/h. Data were collected as previously described in Yang et al. (2022a) and Yang et al. (2022a). The noise measurement instrument is a precision noise level meter, a model AWA6291 handheld real-time signal analyzer. The cockpit light is measured with a digital illuminance meter and the cockpit temperature is measured with a resistance thermometer.

In the experiment, five experts in the field of the car were invited to rate the comfort of the interior environment of the car cockpit. They are all able to provide written consent to an informed form. Table 1 contains brief information about them. Combined with expert opinions, the comfort evaluation scale is shown in Table 2.

**Table 1:** Brief information of experts.

**Expert number**	**Name**	**Gender**	**Position**	**Field of work**
Expert I	Qiping Chen	Male	Professor, East China Jiaotong University, Ph.D.	Intelligent Vehicles
Expert II	Yanli Yin	Female	Associate Professor, Chongqing Jiaotong University, PhD	Intelligent Vehicles
Expert III	Xiaoliang Pan	Male	Senior Engineer, Changan Automobile Company	Vehicle Engineering
Expert IV	Peilong Cheng	Male	Engineer, Tesla Motors	Intelligent Vehicle
Expert V	Yanhong Yang	Female	Senior Engineer of Auto Electric Control Company	Auto Electric Control

**Table 2:** Comfort evaluation grading standard.

**Comfort evaluation level**	**Scoring range**
Intolerable	[0,2]
Very uncomfortable	(2,4]
Uncomfortable	(4,6]
Slightly uncomfortable	(6,8]
Comfortable	(8,10]

With the principle of the single factor variable, the sound decibels, light illumination, and temperature in the car were changed respectively to explore the changing rules of comfort. The corresponding mathematical model is obtained by fitting the experimental data as shown in Table 3.

**Table 3:** Single indicator mathematical model.

• *y*_1_ represents the evaluation value of cockpit noise and vibration comfort; *N* represents the evaluation value of noise obtained by using sound level A as the evaluation method, in dB(A).	
• *y*_2_ represents the evaluation value of cockpit optical environment comfort; *C* stands for illuminance in the cockpit, in lx.	
• *y*_3_ represents the evaluation value of cockpit thermal environment comfort; *T* stands for cockpit temperature in °C	
**Indicator**	**Mathematical model**
Sound decibels	*y*_1_ = − 0.150*N* + 16.795
Light illumination	*y*_2_ = − 0.000031*C*^2^ + 0.033*C* + 0.597
Temperature	*y*_3_ = − 0.054*T*^2^ + 2.649*T* − 22.856

According to the fitting curves of the acoustic environment, optical environment, and thermal environment, the maximum value of comfort evaluation corresponding to the single environment of the cockpit can be obtained as follows.

• Acoustic environment: (50dB(A), 9.295)
• Optical environment: (532lx, 9.379)
• Thermal environment: (24.5 °C, 9.631)

#### Cockpit comfort evaluation model

Since the influence weight of the acoustic environment, optical environment and thermal environment on cockpit comfort is different, the weight vector *ω* = (0.42, 0.23, 0.35) which represents the influence of the acoustic environment, optical environment, and thermal environment on cockpit comfort is set combined with expert opinions. According to the principle of penalized substitution synthesis, the expression of the cockpit comfort comprehensive evaluation model can be obtained, as shown in Eq. (1) below.

(1) \begin{eqnarray*}Y=L+[\max \nolimits (0,{y}_{1})-L]^{0.42}[\max \nolimits (0,{y}_{2})-L]^{0.23}[\max \nolimits (0,{y}_{3})-L]^{0.35}\end{eqnarray*}

In the above Eq., *Y* is the result of the predicted cockpit comprehensive comfort value; *y*_1_, *y*_2_, and *y*_3_ are the dimensionless functions of the comfort index of noise and vibration environment, optical environment, and thermal environment respectively. *L* is the lower limit of any single evaluation index factor of the cockpit, which is 0 in the cockpit evaluation. Therefore, Eq. (1) can be further simplified to Eq. (2).

(2) \begin{eqnarray*}Y=[\max \nolimits (0,{y}_{1})]^{0.42}[\max \nolimits (0,{y}_{2})]^{0.23}[\max \nolimits (0,{y}_{3})]^{0.35}\end{eqnarray*}

When the cockpit comfort evaluation model is used to evaluate the cockpit comfort, it is stipulated that if the comfort score *Y* > 8, the cockpit comfort is considered good and should be maintained. If the comfort score is *Y*<8, it is considered that the cockpit comfort needs to be adjusted.

### Identify the optimal strategy for cockpit comfort adjustment

After evaluating the comfort of the car cockpit, if the comfort score *Y*<8 at a certain time, the comfort of the car cockpit should be adjusted. If the influence degree of three indicators on cockpit comfort at a certain time can be determined, the optimal strategy of cockpit comfort adjustment can be obtained.

### Cockpit comfort adjustment experiment

In this experiment, 90 groups of common environmental conditions in the cockpit were randomly established. After evaluation by the comfort evaluation model, 80 groups of conditions with comfort score *Y*<8 were obtained. The 80 groups of cockpit environment conditions were used for the experiment. After five experts experienced the 80 groups of cockpit environment conditions, the optimal cockpit adjustment strategy was given for the corresponding conditions. Some experimental results are shown in Table 4.

**Table 4:** Cockpit comfort adjustment partial data experimental results. The ‘1, 2, and 3′in column 6 of the table respectively represent experts’ suggestions to adjust the acoustic environment, optical environment, and thermal environment.

**Number**	**Acoustic environment** **dB(A)**	**Optical environment** **lx**	**Thermal environment** °C	**Model comprehensive evaluation value**	**Experts recommend adjustment strategy**
1	67.2	454.3	15.3	6.52	3
2	69.5	1029.2	34.4	4.13	2
3	80.3	999.6	33.8	4.21	2
4	75.4	1008.7	25.3	5.49	2
5	72.4	650.5	36.3	4.57	3
6	68.2	868.5	36.3	4.33	3
	⋮	⋮	⋮	⋮	⋮
86	76.3	450.3	36.6	4.10	3
87	84.4	650.5	36.3	3.93	1
88	70.5	880.6	21.8	6.98	2
89	67.6	985.1	18.8	5.88	2
90	68.2	674.6	23.5	8.00	–

### Optimal state distance

To measure the degree of influence of the three indicators of the acoustic environment, optical environment, and thermal environment on the comfort of the cockpit at a certain time, the distance of an indicator from its optimal state value is used to reflect the necessity of adjustment of the indicator, to propose the optimal adjustment strategy of cockpit comfort. In this article, an optimal state distance (OSD) theory is defined as follows.

**Definition:** Assume that *S* = (*s*_1_, *s*_2_, …, *s*_*n*_) is a feature vector representing an index, where *s*_1_, *s*_2_, …, *s*_*n*_ are *n* attribute vectors constituting the working condition environment. *P* = (*p*_1_, *p*_2_, …, *p*_*n*_) is the feature vector representing the optimal state of the index, where *p*_1_, *p*_2_, …, *p*_*n*_ represents the *n* attribute vectors constituting the optimal state of the index. OS distance is used to measure the distance between an index and its optimal state, as shown in Eq. (3). The larger the OS distance value, the farther an indicator deviates from its optimal state value.

(3) \begin{eqnarray*}D(S,P)=\sqrt{{ \left( \frac{{s}_{1}}{{p}_{1}} -1 \right) }^{2}+{ \left( \frac{{s}_{2}}{{p}_{2}} -1 \right) }^{2}+\cdots +{ \left( \frac{{s}_{n}}{{p}_{n}} -1 \right) }^{2}}\end{eqnarray*}

To make the distance Eq. more universal in engineering applications, the modified parameter *K* is introduced, and Eq. (4) is obtained.

(4) \begin{eqnarray*}D(S,P)=K\sqrt{{ \left( \frac{{s}_{1}}{{p}_{1}} -1 \right) }^{2}+{ \left( \frac{{s}_{2}}{{p}_{2}} -1 \right) }^{2}+\cdots +{ \left( \frac{{s}_{n}}{{p}_{n}} -1 \right) }^{2}}\end{eqnarray*}

In Eq. (4), the correction parameter *K* ∈ (0, 1).

### The optimal cockpit adjustment strategy is identified by OSD

**Figure 4:**
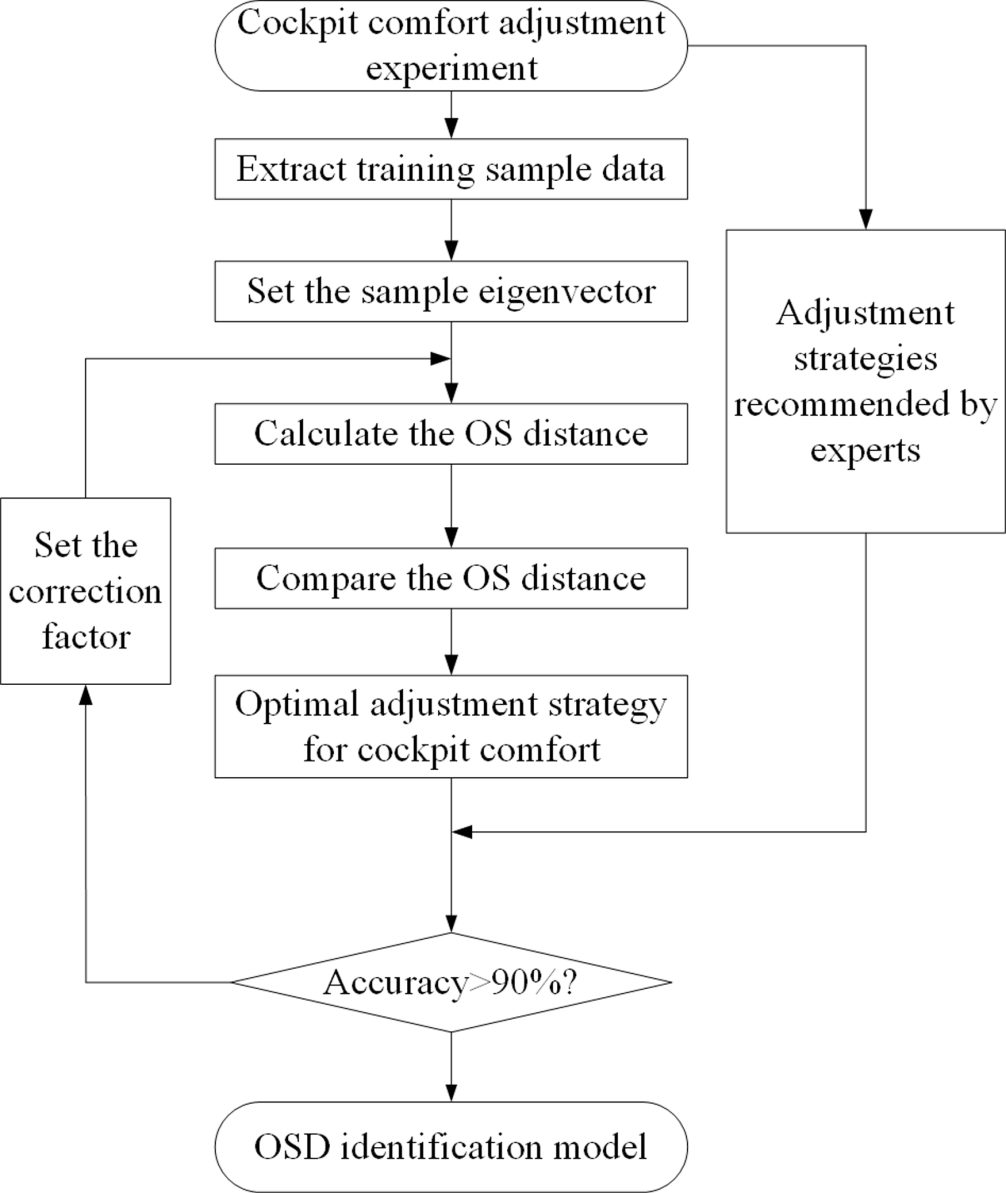
OSD model training process.

To propose the optimal cockpit adjustment strategy, it is necessary to identify the indexes that most affect cockpit comfort in the acoustic environment, optical environment, and thermal environment. In this article, the optimal state distance is used to identify. There are three steps to establish the OSD model. The identification results of the established model are compared with the adjustment target suggested by experts. If the correct rate is greater than 90%, it means that the model is successfully established. The specific flow of this model is shown in Fig. 4.

**Step 1.** Set the feature vector and calculate the OS distance of each indicator

The physical parameter value and the single-factor comfort evaluation value of each index are taken as the two attribute vectors that constitute the characteristic vector of the index. For example, the characteristic vector of the acoustic environment: *S*_*A*_ = (*s*_*A*1_, *s*_*A*2_), where *s*_*A*1_ is the physical parameter of noise, the unit is dB(A), and the *s*_*A*2_ is the comfort evaluation value of the acoustic environment; the optimal state characteristic vector of the acoustic environment: *P*_*A*_ = (*p*_*A*1_, *p*_*A*2_), where *p*_*A*1_ is the optimal state value of noise and its value is 50 dB(A). *p*_*A*2_ is the optimal comfort evaluation value of the acoustic environment and its value is 9.295.

Therefore, the OS distance of the acoustic environment under a certain cockpit operating condition is shown in Eq. (5).

(5) \begin{eqnarray*}{D}_{A}({S}_{A},{P}_{A})={K}_{A}\sqrt{{ \left( \frac{{s}_{A1}}{{p}_{A1}} -1 \right) }^{2}+{ \left( \frac{{s}_{A2}}{{p}_{A2}} -1 \right) }^{2}}\end{eqnarray*}

Similarly, the OS distance of the optical environment and the thermal environment under a certain cockpit working condition *D*_*O*_, *D*_*T*_ as shown in Eqs. (6) and (7).

(6) \begin{eqnarray*}{D}_{O}({S}_{O},{P}_{O})& ={K}_{O}\sqrt{{ \left( \frac{{s}_{O1}}{{p}_{O1}} -1 \right) }^{2}+{ \left( \frac{{s}_{O2}}{{p}_{O2}} -1 \right) }^{2}}\end{eqnarray*}

(7) \begin{eqnarray*}{D}_{T}({S}_{T},{P}_{T})& ={K}_{T}\sqrt{{ \left( \frac{{s}_{T1}}{{p}_{T1}} -1 \right) }^{2}+{ \left( \frac{{s}_{T2}}{{p}_{T2}} -1 \right) }^{2}}\end{eqnarray*}

**Step 2.** Set the correction factor to optimize the OS distance

For the OS distance to reflect the expert’s adjustment recommendations more comprehensively, the correction parameters need to be set. The weight vector *ω* = (0.42, 0.23, 0.35) representing the effects of the acoustic environment, optical environment, and thermal environment on cockpit comfort is introduced above, that is, the weights *ω*_*A*_, *ω*_*O*_ and *ω*_*T*_ of the influence of the acoustic environment, optical environment and thermal environment on cockpit comfort are 0.42, 0.23, and 0.35, respectively. However, considering that the current car in the acoustic environment, optical environment, and thermal environment adjustment difficulty is different, and experts have different adjustment tendencies for the three indicators; therefore, it is necessary to introduce a coefficient *θ* that can represent the expert adjustment tendency, combined with expert opinions, set the expert adjustment tendency coefficients *θ*_*A*_, *θ*_*O*_ and *θ*_*T*_ corresponding to the acoustic environment, optical environment, and thermal environment to 0.27, 0.35, and 0.38 respectively.

Set the correction factor *K* of the OS distance of a certain indicator of the cockpit as shown in Eq. (8).

(8) \begin{eqnarray*}K=\omega \times \theta \end{eqnarray*}

The correction coefficients corresponding to the acoustic environment, optical environment, and thermal environment are calculated:

\begin{eqnarray*}{K}_{A}& ={\omega }_{A}\times {\theta }_{A}=0.1134 \end{eqnarray*}

\begin{eqnarray*}{K}_{O}& ={\omega }_{O}\times {\theta }_{O}=0.0805 \end{eqnarray*}

\begin{eqnarray*}{K}_{T}& ={\omega }_{T}\times {\theta }_{T}=0.1330 \end{eqnarray*}

Define the set *U* = *D*_1_, *D*_2_, *D*_3_, where *D*_1_, *D*_2_, *D*_3_ can be obtained by Eq. (9).

\begin{eqnarray*}{D}_{1}& & =\max \nolimits \left\{ {D}_{A},{D}_{O},{D}_{T} \right\} \end{eqnarray*}

(9) \begin{eqnarray*}{D}_{3}& & =\min \nolimits \left\{ {D}_{A},{D}_{O},{D}_{T} \right\} \end{eqnarray*}

\begin{eqnarray*}{D}_{2}& & ={C}_{U}({D}_{1}\cup {D}_{3}) \end{eqnarray*}

By calculating the OS distance of the three indicators, the index with the largest output distance is the goal that urgently needs to be adjusted at a certain time in the cockpit, that is, it is *j*_1_ for the optimal adjustment strategy of cockpit comfort. *j*_1_ is expressed by Eq. (10).

(10) \begin{eqnarray*}{j}_{1}= \left\{ \begin{array}{@{}c@{}} \displaystyle A\\ \displaystyle O\\ \displaystyle T \end{array} \begin{array}{@{}c@{}} \displaystyle {D}_{1}={D}_{A}\\ \displaystyle {D}_{1}={D}_{O}\\ \displaystyle {D}_{1}={D}_{T} \end{array} \right. \end{eqnarray*}

where *A*, *O*, and *T* represent that the cockpit should adjust the acoustic environment, optical environment, and thermal environment, respectively.

**Step 3.** Model accuracy optimization.

The defined *ɛ* is the OS distance difference, which is calculated as shown in Eq. (11). Set the accuracy of the distance difference *ɛ*_*o*_ = 0.01.

(11) \begin{eqnarray*}={D}_{1}-{D}_{2}\end{eqnarray*}

When calculating the OS distance, if it is *ɛ* ≤ *ɛ*_*o*_, it is considered that the *D*_1_ and *D*_2_ are almost equal, and based on expert suggestions, the party with the largest expert adjustment tendency coefficient *θ* is preferred as the optimal cockpit adjustment strategy *j*_2_. Since the expert adjustment tendency coefficients *θ*_*A*_, *θ*_*O*_ and *θ*_*T*_ set above are 0.27, 0.35, and 0.38, respectively, *θ*_*T*_ > *θ*_*O*_ > *θ*_*A*_ can be obtained. *j*_2_ is expressed by Eq. (12).

(12) \begin{eqnarray*}{j}_{2}= \left\{ \begin{array}{@{}l@{}} \displaystyle O {D}_{3}={D}_{T} \\ \displaystyle T \left\{ \begin{array}{@{}l@{}} \displaystyle \begin{array}{@{}c@{}} \displaystyle {D}_{3}={D}_{O}\\ \displaystyle {D}_{3}={D}_{A} \end{array} \end{array} \right. \end{array} \right. \end{eqnarray*}

In Eq., *O* and *T* represent that the cockpit should be adjusted to the optical environment and thermal environment, respectively.

**Step 4.** The final cockpit optimal adjustment strategy identification model

From the above derivation, combined with the analysis of the optimal cockpit adjustment strategy *j*_1_, *j*_2_, the final cockpit optimal adjustment strategy *J* is obtained as shown in Eq. (13).

(13) \begin{eqnarray*}J= \left\{ ~\begin{array}{@{}c@{}} \displaystyle {j}_{1}\\ \displaystyle {j}_{2} \end{array}~\begin{array}{@{}c@{}} \displaystyle \gt {}_{o}\\ \displaystyle \leq {}_{o} \end{array}~ \right. \end{eqnarray*}

**Figure 5:**
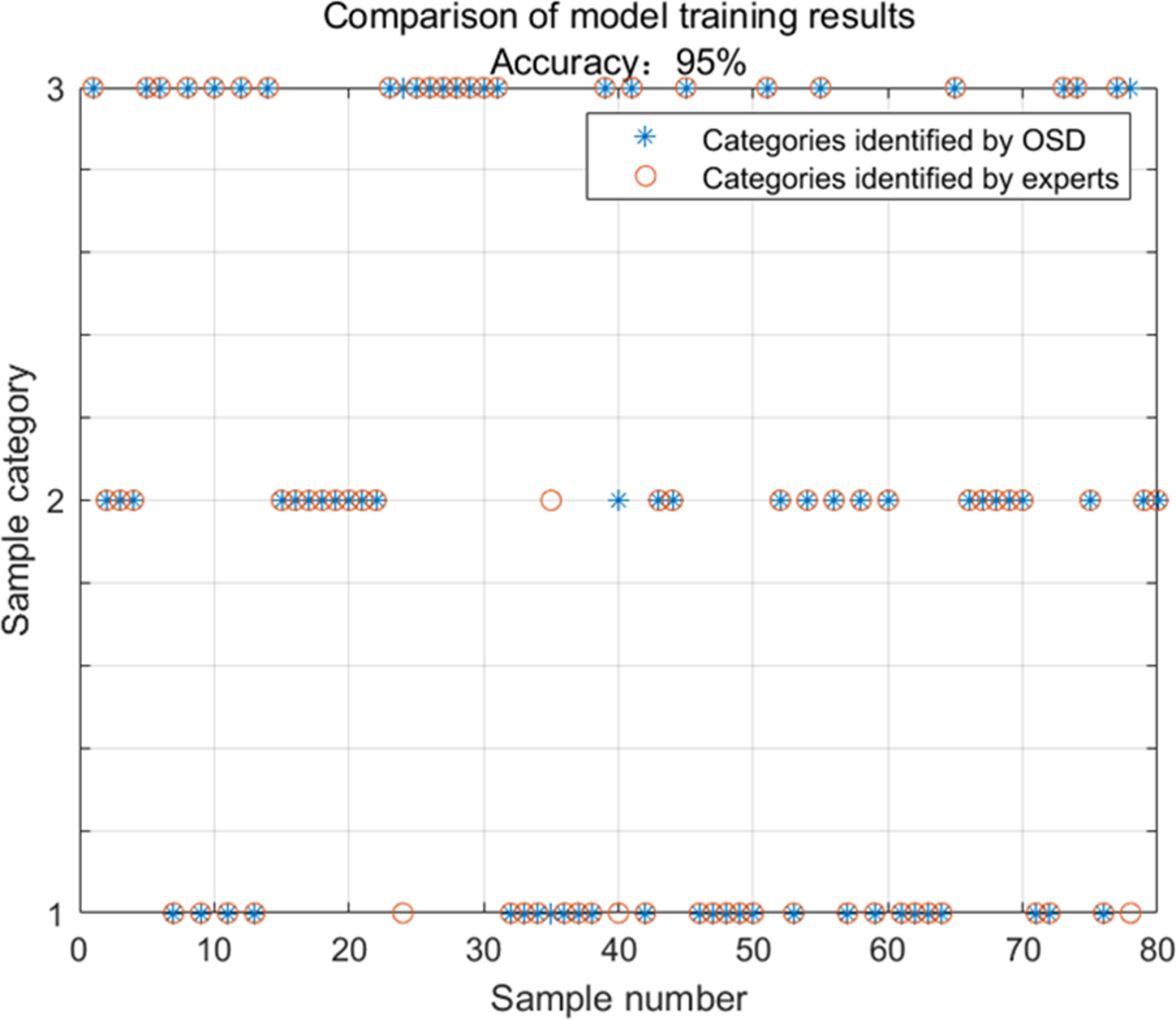
Model identification results.

The model was used to identify 80 groups of test conditions that needed to be adjusted for cockpit comfort, and the optimal adjustment strategy of cockpit comfort based on OS distance was obtained, which was compared with the adjustment strategy suggested by experts. The results are shown in Fig. 5.

Comparing the optimal comfort adjustment strategy *J* obtained by OSD with the expert-suggested adjustment strategy, it can be seen that the number of samples consistent with the expert’s recommendation according to the comfort adjustment strategy obtained by OSD is 76, and the total sample is 80, with a consensus rate of 95%. It can be seen that the model is successfully established and has a good identification effect, which can obtain the optimal strategy for cockpit comfort adjustment.

## Results and Discussion

### Cockpit comfort evaluation

In this article, starting from the three indicators of the acoustic environment, optical environment, and thermal environment that affect the comfort of the car cockpit, the car cockpit comfort evaluation experiment is carried out. The cockpit evaluation experiment created in this article simulates the real occupant riding state and fully considers the riding experience of experts. Through the fitting analysis of the experimental data, a single index comfort evaluation model is obtained. Then, by combining the comfort evaluation model of a single index, the cockpit comfort evaluation model was established. The model can calculate the comfort evaluation value *Y* of the current cockpit environment through the physical parameter values of sound decibels, light illuminance, and temperature in a certain cockpit working environment. When the cockpit comfort evaluation value is *Y* > 8, it is considered that the comfort of the cockpit comfort is good; Conversely, it is believed that the comfort of the cockpit still needs to be improved.

 This article has a comprehensive evaluation of car cockpit comfort, is a comprehensive evaluation of comfort, and has certain innovative value in engineering applications. With the innovation and development of science and technology, people’s cockpit comfort requirements will become higher and higher. In addition to the acoustic environment, optical environment, and thermal environment, more indicators will be added, and this article provides a reference direction for future cockpit comfort evaluation.

### Identify optimal adjustment strategies for cockpit comfort

#### Model validation

**Figure 6:**
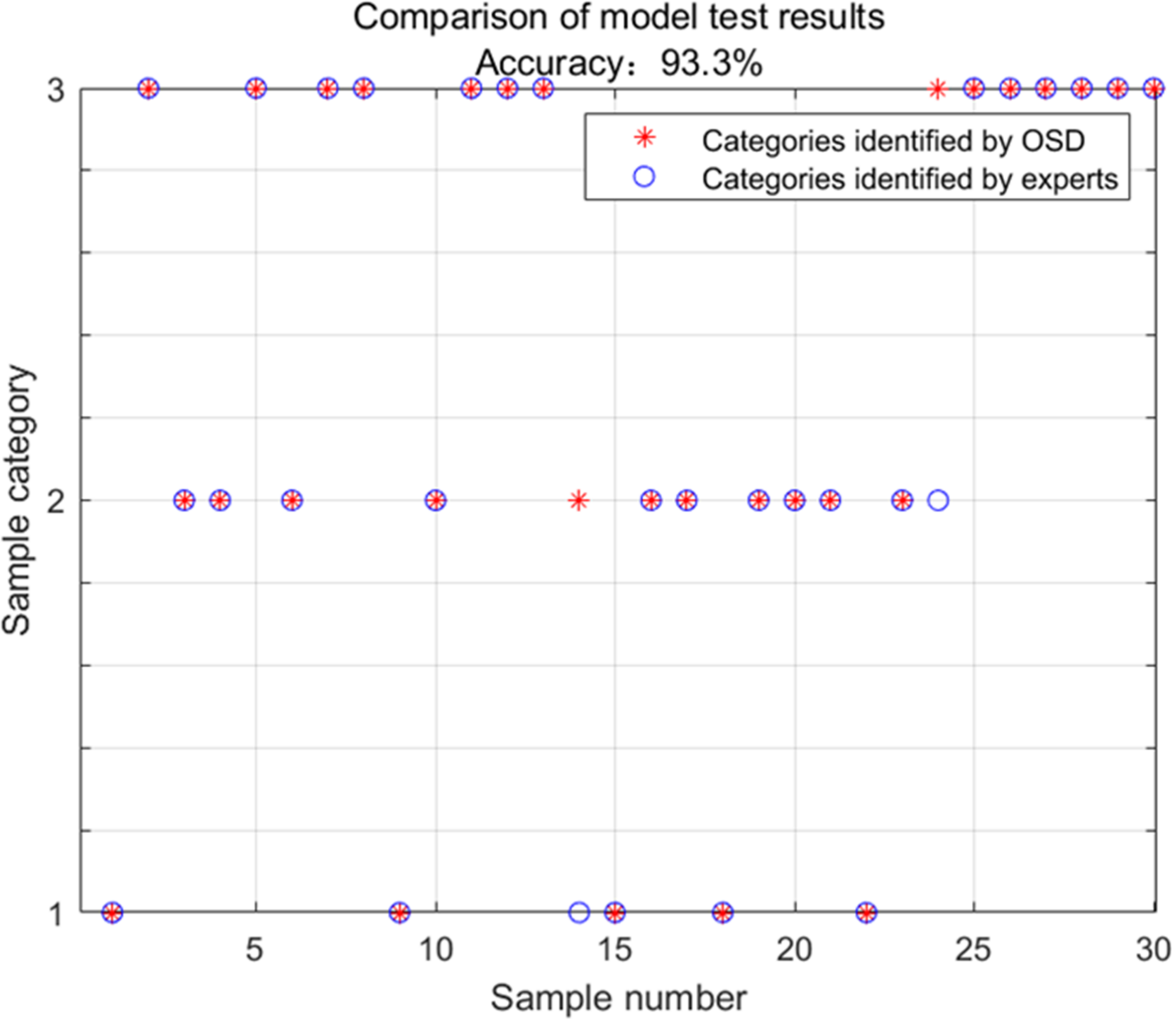
Model validation results.

To verify the accuracy and usability of the model, 30 different cockpit environmental conditions were randomly set, and the comfort score of each working condition was obtained from the cockpit comfort evaluation model, and the cockpit working conditions that needed to be adjusted were compared with OSD identification and machine learning identification. The identification results are shown in Fig. 6. Table 5 has the specific experimental information for each group of experiments.

**Figure 7:**
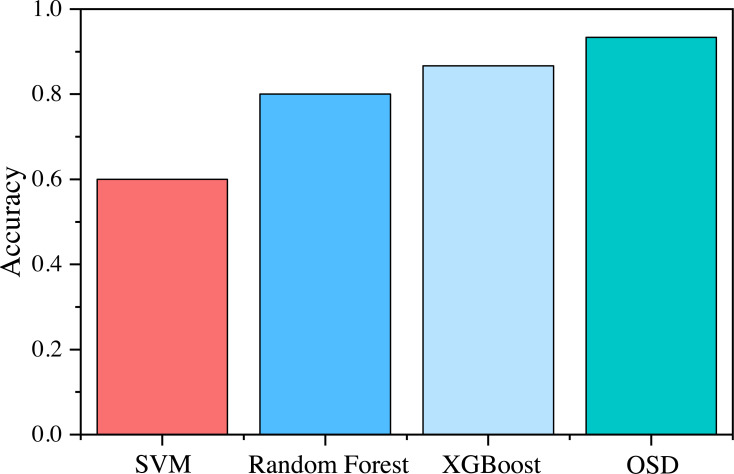
Accuracy comparison.

Combined with Fig. 6 and Table 5, it can be seen that the accuracy rate of the cockpit environmental adjustment strategy identified by OSD identification is 93.3%, and the accuracy rate is higher than all machine learning methods. The effect of OSD identification is more prominent (as shown in Fig. 7).

#### Model analysis

After the evaluation of cockpit comfort is completed, it is necessary to adjust the cockpit where comfort will be poor. In a certain cockpit working environment, it is often necessary to adjust the indicators that most affect the comfort of the cockpit to have a better adjustment effect. In this article, an OSD method is introduced to measure the distance that each indicator deviates from its optimal state value. Based on the OSD, the correction parameters are introduced in combination with the specific engineering application environment, and the optimal cockpit adjustment strategy identification model is established. Combined with the cockpit comfort adjustment experiment, it is known that the accuracy of the model is greater than 90%, and it has a good effect. Through this model, the indicators that have the greatest impact on cockpit comfort in a certain cockpit working environment can be identified, and the corresponding actuator can be adjusted to improve cockpit comfort.

**Table 5:** The model validates the data information. The ‘1, 2, and 3′in columns 6 to 10 of the table respectively represent optimal adjustment strategies.

• 1—Acoustic environment									
• 2—Optical environment									
• 3—Thermal environment									
**Number**	**Acoustic environment** **dB(A)**	**Optical environment** **lx**	**Thermal environment** °C	**Model comprehensive evaluation value**	**Experts recommend adjustment strategy**	**OSD identification adjustment strategy**	**SVM identification adjustment strategy**	**Random Forest identification adjustment strategy**	**XGBoost identification adjustment strategy**
1	82.5	450.3	30	6.44	1	1	3	1	1
2	72.3	868.9	35.4	4.80	3	3	2	3	3
3	70.5	880.6	21.8	6.98	2	2	1	1	2
4	67.6	985.1	18.8	5.88	2	2	2	2	2
5	68.2	674.6	33	6.70	3	3	3	3	3
6	66.4	1011.7	27.6	5.86	2	2	2	2	2
7	63.5	698.5	12	3.96	3	3	1	2	3
8	77.2	582.6	36.4	4.27	3	3	3	3	3
9	79.6	654.3	30.8	6.50	1	1	3	1	1
10	59.6	939.9	26.8	7.24	2	2	2	2	2
11	77.6	518.2	34.3	5.63	3	3	3	3	3
12	64.8	495.4	37	4.09	3	3	3	3	3
13	69.4	599.6	33.9	6.33	3	3	3	3	3
14	79.5	999.3	30	5.03	1	2	2	2	2
15	84.2	422.7	22.6	6.62	1	1	3	1	1
16	78.2	860.3	33.8	5.25	2	2	1	3	3
17	69.1	949.4	23.5	6.62	2	2	2	2	2
18	76.5	877.5	26.4	6.60	1	1	1	1	1
19	71.5	211.7	27.8	7.01	2	2	3	2	2
20	67.9	985.1	18.8	5.87	2	2	2	2	2
21	62.6	939.9	26.8	7.06	2	2	2	2	2
22	74.5	799.3	30	6.73	1	1	1	1	1
23	64	927.9	31.2	6.48	2	2	2	2	2
24	64.2	931.1	34.5	5.35	2	3	2	2	3
25	78.3	856.8	12.4	3.60	3	3	1	1	3
26	76.2	1004.2	36.6	3.04	3	3	2	3	2
27	82.6	908.2	36.9	3.01	3	3	1	3	3
28	67.4	444.9	36.4	4.73	3	3	3	3	3
29	77.5	644.2	34.8	5.34	3	3	3	3	3
30	56	489.1	33.1	7.49	3	3	3	2	3

 The OSD method has excellent generalization performance and low computational complexity, which can also ensure high recognition accuracy when the number of target categories to be identified increases. Therefore, this method can also be applied to more complex engineering practices.

### Dynamic adjustment for cockpit comfort

**Figure 8:**
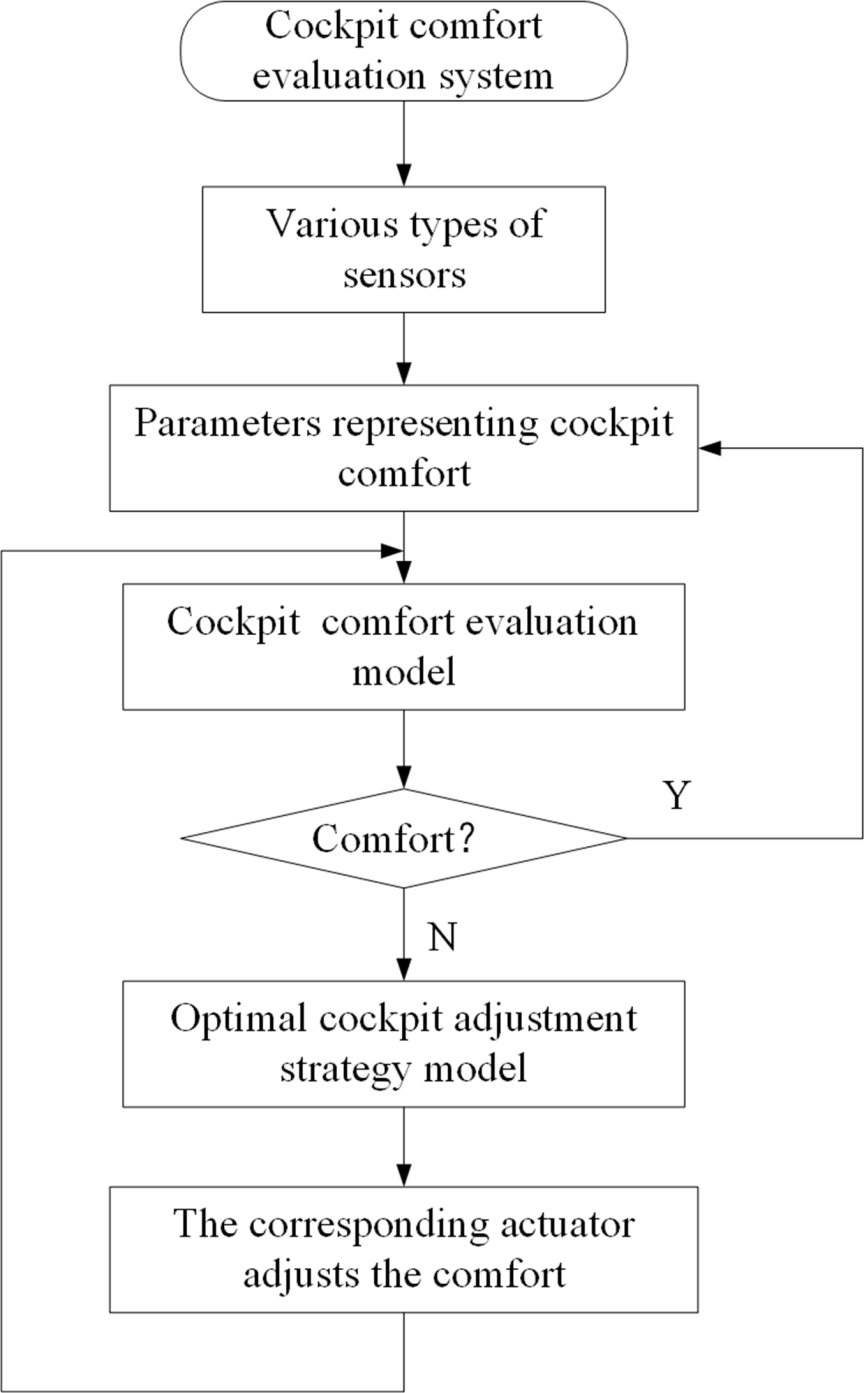
Dynamic detection and adjustment of cockpit comfort.

In the process of driving the car, the environment in the cockpit of the car is constantly changing, so the cockpit comfort is also constantly changing. After the evaluation of cockpit comfort and the identification of optimal adjustment strategies are realized, the cockpit comfort can be monitored and adjusted (as shown in Fig. 8).

 In specific engineering practice, a cockpit comfort evaluation system can be installed in the ECU of a vehicle. The sensors collect the specific physical parameters of the acoustic, light and thermal environments and transmit them to the ECU for processing, and the ECU obtains the comfort evaluation value of the cockpit at a certain moment according to the cockpit comfort evaluation model. Then, according to the cockpit comfort optimal adjustment strategy model, the optimal control strategy is obtained, and the corresponding actuator is operated to adjust the comfort level. This facilitates the dynamic adjustment of the cockpit comfort to ensure occupant comfort.

## Conclusions

With the rapid development of the automotive industry, cockpit comfort has become the standard for judging the quality of cars, and people’s requirements for cockpit comfort are getting higher and higher. The cockpit comfort evaluation model established in this article can comprehensively evaluate the acoustic environment, optical environment, and thermal environment of the cockpit, and can give accurate comfort evaluation values in a certain cockpit working environment.

During the driving of the car, the cockpit microenvironment will continue to change, and the comfort will also change, so it is difficult to maintain good comfort in the intelligent cockpit microenvironment of the car. When the overall comfort of the cockpit is reduced and the occupants feel uncomfortable, the cockpit comfort needs to be adjusted. However, the main causes of discomfort in the cockpit microenvironment may be different, and how to adjust the cockpit comfort is particularly important. In this article, the optimal adjustment strategy of the cockpit working condition is obtained with the help of the OSD method, to facilitate the timely adjustment of the corresponding actuator and realize the dynamic monitoring and adjustment of cockpit comfort.

This article is highly innovative in the evaluation and adjustment of cockpit comfort, which can be widely used in engineering control-related fields and has far-reaching significance.

### Limitations and future work

#### Model limitations

(1) This article only considers the three indicators of the acoustic environment, optical environment, and thermal environment that affect the comfort of an intelligent cockpit, with the development of intelligent vehicles, indicators such as human–computer interaction environment comfort should become part of the cockpit comfort evaluation system.

(2) Five experts in the automotive field were invited to this experiment, and due to the small number of participants in the experiment, gender, age, and other factors may affect the experimental results.

(3) The experiment was carried out in southwest China, where the temperature of the four seasons is relatively high, and the influence of region, cultural customs, and ethnicity has a certain influence on the experimental results.

(4) Only one car was used for this experiment, which may have an impact on the robustness of the model.

### Future work

In the future, our team will work to establish a more comprehensive and complete cockpit comfort evaluation system, establish more detailed and comprehensive experiments by increasing the number and types of car models, optimize the cockpit comfort evaluation model, improve the cockpit optimization adjustment strategy model based on optimal state theory, and strive to achieve dynamic adjustment of cockpit comfort and improve the occupant’s riding experience.

##  Supplemental Information

10.7717/peerj-cs.1324/supp-1 Supplemental Information 1 Single indicator comfort experiment data

10.7717/peerj-cs.1324/supp-2 Supplemental Information 2 Cockpit identification experiment data

## Additional Information and Declarations

### Data Availability

The following information was supplied regarding data availability: The raw measurements are available in the Supplemental Files.
